# Patients' experiences of the decision‐making process for clinical trial participation

**DOI:** 10.1111/nhs.12933

**Published:** 2022-03-21

**Authors:** Trine A. Gregersen, Regner Birkelund, Maiken Wolderslund, Karina Dahl Steffensen, Jette Ammentorp

**Affiliations:** ^1^ Centre for Research in Patient Communication Odense University Hospital Odense Denmark; ^2^ Department of Clinical Research Faculty of Health Sciences University of Southern Denmark Odense Denmark; ^3^ Department of Regional Health Research Faculty of Health Sciences University of Southern Denmark Odense Denmark; ^4^ Department of Oncology Lillebaelt Hospital ‐ University Hospital of Southern Denmark Vejle Denmark; ^5^ Center for Shared Decision Making Lillebaelt Hospital ‐ University Hospital of Southern Denmark Vejle Denmark

**Keywords:** clinical decision‐making, clinical trials, health communication, nurse's role, person‐centered care, physician's role, therapies

## Abstract

Clinical decision‐making about participating in a clinical trial is a complex process influenced by overwhelming information about prognosis, disease, and treatment options. The study aimed to explore patients' experiences of the decision‐making process when patients are presented with the opportunity to participate in a cancer clinical trial and to shed light on how patients experience the health communication, the nurse's role, and the physician's role. A qualitative study design was applied. Nine patients with advanced cancer were interviewed after being informed about their treatment options. Data were analyzed using thematic analysis. The results showed that patients made treatment decisions mainly guided by their emotions and trust in the physician. Furthermore, the physicians had a great impact on the decisions, and the nurse's role was associated with conversations about how to manage life. The study highlights the importance of talking about prognosis and addressing the patient's existential issues, particularly in this context of advanced cancer. The study elucidates a need for healthcare professionals to engage in health communication about life when it is coming to an end.

**Key points**

- Patients made clinical trial decisions mainly guided by their emotions and trust in the physician.
- Physician's role had an impact on the decisions. Patients considered the physician to be the expert in the clinical decision‐making and relied on the physician's competences.
- Nurse's role was associated with conversations about existential aspects of living such as how to manage life with advanced cancer.

## 1 INTRODUCTION

Advances in cancer treatment are founded on research that tests new drugs and methods. One of the final stages of this long research process is the oncology clinical trial. In the first phases of these trials, new treatments are tested to determine whether they cause any serious harm, whereas the main purpose of the later phases is to explore the risks and benefits of the new treatment and to determine whether the experimental treatment is better than the standard treatment (National Institutes of Health, 2018).

To protect patients and ensure reliable study results, clinical trials follow strict scientific procedures. One of these procedures is informed consent (Emanuel et al., 2000; WMA, 2013), which aims to inform patients about the research and provide them with voluntary consent to participate in a clinical trial. Healthcare professionals are responsible for ensuring that patients understand the purpose, the procedures, and the potential harms and benefits of their involvement in the trial, and not least of all, the alternatives to participation.

Clinical trials provide both oral and written information and entail a complex and time‐demanding consent process guided by ethical principles to protect the patients involved (World Medical Association, 2013). Decisions about participating in a clinical trial can be particularly difficult because patients will have to choose between a well‐known and accepted standard treatment and experimental treatment whose treatment efficacy and side effects are less documented and might influence the patients' quality of life (Joseph‐Williams et al., 2014; Mills et al., 2006). Furthermore, this decision‐making process takes place at a time when the cancer patient and their relatives are in a highly vulnerable situation because of the life‐threatening nature of the disease (Schaeffer et al., 1996).

The vast majority of research studies have focused on either recruitment or improving consent information in relation to trial participation. Moreover, most studies on patients' experiences about clinical trial decision‐making have been conducted in curative settings, whereas less focus has been on the palliative setting where the cancer is advanced and a cure is no longer possible.

Existing research on clinical trial decision‐making in the context of advanced cancer shows that the desire for curative treatment is the patients' primary motivation for clinical trial participation (Godskesen et al., 2013; Kvale et al., 2010) and that many patients are willing to try anything to achieve this (Harrop, Noble, et al., 2016; Kohara & Inoue, 2010) despite having been informed that the cancer is incurable. The research literature also indicates that patients are aware that they can decline further treatment but they tend to think that their life situation gives them no other choice than to opt for treatment in a clinical trial when offered participation (Moore, 2001).

These findings indicate the complexity of the decision‐making process and that many patients with advanced cancer also feel overloaded with information provided by the healthcare professionals at a difficult and emotional time in life (Harrop, Kelly, et al., 2016; Shannon‐Dorcy & Drevdahl, 2011). However, little is known about the patients' experiences with regard to the decision‐making process.

### 1.1 Aim

This study aimed to explore patients' experiences of the decision‐making process when presented with the opportunity to participate in a cancer clinical trial. The research questions were:

1. How do patients experience the conversation with the healthcare professionals?
2. How do patients experience the role of physicians and nurses in the consultation?
3. How do patients experience the decision‐making process?

## 2 METHODS

### 2.1 Data collection

Patients' experiences were explored through semistructured, in‐depth interviews inspired by Kvale and Brinkmann (Kvale & Brinkmann, 2014) and data were analyzed using thematic analysis inspired by Braun and Clarke (Braun & Clarke, 2006). This study is part of a larger study in which additional data are collected through participant observations of a clinical outpatient setting where trial decisions are made.

Patients were recruited at a public university hospital in Denmark at the Department of Oncology from July 2016 to May 2019. The patient population included were all patients with advanced cancer offered to participate in a clinical trial. A total of 20 patients were approached for study participation and nine patients were interviewed, eight women and one man (Table 1). Before the interview, all patients had already received a consultation with an oncologist and an oncology nurse where they were informed about the cancer having progressed despite current treatment. During the same consultation, the patients were informed about new treatment options, including the possibility of participating in a clinical trial.

**TABLE 1:** Patient characteristics

Participant	Gender	Disease	Age at interview	Treatment decision
Patient 1	Woman	Ovarian cancer	68	Standard
Patient 2	Woman	Breast cancer	55	Trial^a^
Patient 3	Man	Colon cancer	68	Trial
Patient 4	Woman	Ovarian cancer	67	Standard
Patient 5	Woman	Colon cancer	54	Trial
Patient 6	Woman	Colon cancer	54	Trial
Patient 7	Woman	Colon cancer	63	Trial
Patient 8	Woman	Breast cancer	43	Trial
Patient 9	Woman	Breast cancer	66	Trial

^a^ Chose trial but could not be included.

The interviews took place the same day as the consultation and up to 22 days after the consultation where the patients were informed about the clinical trial, most commonly within 2 weeks from the consultation. The interviews lasted between half an hour and 2 h and took place at the patients' home or the hospital, depending on each patient's wishes. Three patients preferred a telephone interview. An interview guide was developed by the first author in cooperation with two of the coauthors, based on participant observation, knowledge of the field, and review of the literature. The interview guide contained open‐ended questions and adjustments to the guide were made after discussions with the coauthors before and after interviews. All interviews were performed by the first author, recorded, and transcribed verbatim.

### 2.2 Analysis

Applying thematic analysis, we searched to discover patterns and characteristics in the interview transcripts using an ongoing iterative process that followed six overall phases of the analysis, inspired by Braun and Clarke (Braun & Clarke, 2006).

1. Reading and rereading the interview transcripts to become familiar with the data and to achieve a general understanding of the data.
2. Coding data by searching through the interview transcripts and identifying all kinds of assertions that might be important in order to investigate the aim of the study. For the organization of data, we used NVivo11 (NVivo., 2015), and the identified codes were highlighted in NVivo.
3. Identifying themes and broader meaning by examining the identified codes. During this process codes were grouped into hierarchical structures and new codes were created to capture the meaning of each of these groups.
4. Continuing theme development with review of the identified themes against the interview transcripts to check that the themes cover the story being told in the interviews.
5. Developing a detailed analysis of each theme to determine the focus and description of each theme. When the final themes were identified, relevant quotes were chosen to illustrate each of the themes.
6. Writing up the results, going beyond the description of the themes, and contextualizing the analysis in relation to existing research literature and relevant theoretical perspectives (which will be unfolded in the discussion section).

### 2.3 Ethical considerations

The study was approved by the Danish Data Protection Agency through the Region of Southern Denmark. According to Danish law, interview studies do not need ethical approval; however, the Regional Scientific Ethical Committees for Southern Denmark was notified. Patients and relatives provided verbal and written informed consent and were informed about their right to withdraw consent.

### 2.4 Rigor

To ensure transparency (Kvale & Brinkmann, 2014), a detailed description of the participants, setting, data collection, and transcribing of data are described in the methods section. The six phases of the analysis are specifically outlined to ensure transparency throughout the data analysis. The study findings were evaluated through validation discussions with coauthors and fellow researchers which occurred throughout the whole data generating process, analysis, and presentation of findings. In this way, the reflections ensured coherence between interviews and interpretation (Kvale & Brinkmann, 2014).

## 3 RESULTS

Four themes were identified and presented in Table 2, which also shows examples of codes leading to the themes. Each theme is described and illustrated with quotes from the interviews.

**TABLE 2:** Themes and examples of codes

Themes	Theme 1	Theme 2	Theme 3	Theme 4
	Specific expectations regarding physician's role and nurse's role	Do I have any other choice?	Information overload in a vulnerable situation	When clinical trial takes center stage
Examples of codes	Role distribution	Fear of the unknown	Information overload	Isolated from context
	Trust	Treatment is hope	fishing for answers	Clinical trial
	Competence	No other choice	Want answers	Referred patients
	Physicians recommendation	Not done with life	A contradictory answer	Who has the responsibility
	Practical information	Hope for a cure	Thorough information	Overview of the course of the disease
	Nurses' role	Control	Shocking information	Being lost

### 3.1 Specific expectations regarding physician's role and nurse's role

It appeared from the interviews that the patients had very specific expectations regarding the respective roles of the physician and the nurse, and they experienced that the physicians and nurses contributed to separate parts of the conversations.

Some patients did not expect the physicians to have the time to talk about other issues than symptoms and treatment. One patient who was referred from another hospital to receive information about experimental treatment said:

It's the first time where I've experienced they went closer… Doctor appointments have just been brief and factual. It is the first time being here with this doctor where we have spent so much time, so it's somehow a different encounter than I've been used to. Of course, it's fine too. I just didn't have the impression that doctors had the time for anything else than to send me further on with some medicine and chemo.

Other patients described how they were fishing for answers about life expectancy and how they tried to ask about prognosis and time left to live. These patients felt that they did not get the answer they wanted at the consultations and experienced that the most frustrating thing about the conversations was not getting a result. To go further into these statements, one patient said: *That's the one thing they can't tell us, whether I'll become cancer‐free*. Another patient said: *I also think, should I ask? I think it is his job too to say how the next course of disease might look*, while one of the other patients talked a great deal about the things that had remained unsaid: *That part… the unmentioned part… maybe that part about how big the chance is that I'll survive this. That's what makes it tough*.

Relational aspects were of great importance for the patients in the decision‐making process. Most patients considered the physician to be the expert in the decision‐making and relied on the physician's competences. One patient said: *It is difficult for me to choose because you don'’t know what is best. The physician must know that*. Similar to this, several patients expressed the same viewpoint with comments like: *I don'’t understand it. I am not a physician*. In addition to the perception of the physician as the expert, patients' decisions about whether or not to participate in a trial were influenced by their trust in the physician. Many patients were willing to choose experimental treatment because they felt safe at the hospital and trusted that the physician would only suggest the treatment if he/she had an idea that it might help them. One patient explained: *Yes, they would not guide you if they knew in advance that it would be of no good or not have any effect*. These patients who entrusted the decision to the healthcare professionals followed the physicians' recommendation when they sensed that the physician recommended one of the treatment options. *Yes, if the physician believes that's what you should do, then I just follow it. I always adopt what the physician says because I have no other options*.

Contrary to trusting the physician, in some cases where something went wrong in the information about the experimental treatment which led to confusion about schedule, examinations, or side effects, patients said that they felt unsafe about their decision to participate in the trial and that they considered dropping out.

Patients did not talk much about the nurses or their expectations of the role of the nurse. Several patients stated that the nurses' presence during the consultation was not of great importance and that it seemed to play a minor or supportive role in the decision‐making process. This might to some extent be explained by the fact that the present study focuses on patients' experiences of treatment decision‐making and diagnostic and medical decisions are a part of the physicians' field of work. One patient expressed:

I did not think much about the nurse sitting there. I was mostly concentrating on the doctor. The nurse had the practical information. Something about hair loss and wigs, but she was not allowed to recommend anything.

In contrast to this, a few patients talked differently about the nurse's role at the consultations. These patient experiences seemed to be linked with their relationship to the nurse whereby the nurse knew them well and had helped them through existential crises. Moreover, several patients mentioned that the nurses talked with them about how they managed life with cancer while giving them treatment at the outpatient clinic after the consultations. One patient said:

When I've had chemo it's the nurse who sits next to me and then we spend maybe half an hour, an hour and a half … and talk about how one's psychical condition is, how to manage things, so it's mostly while receiving treatment and it has been nurses who have talked with you. They have been really great at it, I think.

Moreover, in talking about the nurses' role in general, several of the patients' statements reflected that they experienced conversations with the nurse to focus more on them as a person and not a patient. As an example, one of the patients said: *When you get such a message, you can't think of anything else* … *at a time like that, you should have half an hour with a nurse where the focus was only on me*.

### 3.2 Do I have any other choice?

Patients talked about their reasons for choosing experimental treatment as a way to avoid the fear of the unknown. They preferred to be in treatment to avoid the scary feeling that emerged when they were not receiving treatment. Patients stated that they knew that something was going on in their body and that the cancer was growing when they did not receive treatment, which made it hard to be between prior treatment and waiting for a new treatment to begin. Receiving treatment made the patients feel like they had control over the disease and when told that their present treatment was no longer effective, they demanded a new treatment plan. In relation to this, patients felt that receiving treatment meant hope to live much longer, and some of the patients hoped the experimental treatment would cure the cancer.

The patients had other reasons for deciding whether or not to participate in a clinical trial, such as wanting to help others in the future by contributing to research in cancer treatment, and some liked the extra attention as a consequence of participating in a trial.

Some patients who had no other treatment options felt they had no other choice but to try the experimental treatment as part of the trial enrolment. One patient said: *I do not really have any other choice. They do not have other options at the other hospital. They have tried what they have. So, there is no choice for me*. Similar to this example, several of the patients talked about having no choice and often repeated that they decided to participate in the trial because it was their only choice or because they felt they had no choice. For most of the patients, standard treatment was also an option but the interviews revealed that many patients did not experience this as a real option because their physician had told them that standard treatment would not have a major effect on their disease. Neither was the possibility of “no treatment” perceived as a real option because it felt like giving up on life. Only one of the patients stated “no treatment” as a possibility when she was talking about treatment options. Common to these patients who made the decision with the feeling of having no choice was that they talked about the decision as an obvious choice. As the following example illustrates, some of the patients expressed that they chose experimental treatment because they were not done with life: *No, I don't think I'm completely finished with life yet. I'd like to give it a try, all the options I have altogether*.

One patient said that she would choose experimental treatment if that were her only treatment option. This was also the case for some of the other patients, indicating they were willing to try anything to survive. Moreover, patients who had to choose between experimental treatment and standard treatment sometimes needed more time to think about their decision. For instance, one patient explained: *It was not something I dared to say yes to. I had to read more about it*. The statements from these patients implied that some of them did in a way feel like they had a choice, indicating that there are individual circumstances that apply for the patient and their perception of the decision.

### 3.3 Information overload in a vulnerable situation

The patients expressed satisfaction with the information they received and described both the oral and written information as being thorough and exhaustive. However, several patients expressed that they received too much information, especially referring to oral information and described that it felt like an information overload being in that particularly vulnerable situation. For example, one patient said: *In general, I got a whole lot of information and I couldn't quite handle it all*.

Some patients experienced receiving shocking information at the consultation, referring to cases where the physician had talked with them about life and death. Some patients stated that they did not expect that to be a part of the consultation; their expectation of the consultation was mostly to be informed about treatment. One patient said: *We were a little shocked by the talk about me having considered my own death and all that*. Similar to this example, several patients expressed it was difficult for them to talk about life coming to an end.

Commonly, patients were confused about the physician's message. One of the patients called it a contradictory answer because the physician had told her to maintain the hope of living many more years and at the same time said that she had to be aware that if something needed to be taken care of, she should do it soon:

The part about me telling the doctor that I hoped for 10 more years, and he told me that it was a good starting point. Then I'm kind of in doubt. What should I make of it? Is it because you are saving a hope or is it because you… But he also said that maybe there were some things to take care of, for instance a will and such. So that's like a contradictory answer.

However, when talking with the patients using statements like the aforementioned, the patients often expressed an understanding of the physicians challenges in making customized treatment information because the individual patients’ considerations varied in the treatment decisions.

### 3.4 When clinical trial information takes center stage

Some of the patients were referred from other hospitals for a specific experimental treatment that was not offered at their district hospital. In these cases, patients received information about the trial and received experimental treatment (if they chose to participate in the trial) at another hospital than their district hospital. This made them experience that the information they received was focused solely on the trial. Some of the patients stated they preferred it that way and said they did not come to talk about their feelings. Other patients said that because they chose experimental treatment at another hospital district, they felt that neither the hospital where they received experimental treatment nor the hospital where they had earlier received treatment had a full overview of the course of the disease. One patient said:

I feel that I have fallen between the cracks because I am now referred to X Hospital but I belong in Y Hospital. Who is the one responsible for consistency in my treatment? No one is… so who is it that has the common thread in my treatment if I suddenly get really sick? No one has. It has come to my attention that it is a side effect, so to speak, of being in such a trial.

This meant these particular patients experienced the information as being isolated from the context of having advanced cancer, and they reported that they did not experience that their lives as patients were taken into account. These experiences were not to the same extent shared by the patients whose decision‐making about experimental treatment took place at their local district hospital.

## 4 DISCUSSION

The present study provides insight into patients’ experiences of the complex process of making decisions about clinical trial participation in the context of advanced cancer. The findings revealed that patients’ decisions were guided mainly by their emotions (e.g., fear, hope, trust, and having no choice). Receiving treatment gave the patients a feeling of hope and a sense of having control over the disease and their own body. Furthermore, they often chose to receive treatment to avoid feelings of fear and a feeling of being out of control in managing the disease. These findings match existing research literature showing that patients with incurable cancer often choose to participate in a clinical oncology trial because of the hope for a cure for cancer (Gregersen et al., 2019). Moreover, other studies show that being in treatment can be a way for patients to manage the feeling of fear when having cancer and a way to enable hope (Godskesen et al., 2013; Quinn et al., 2012). Accordingly, patients have described how the feeling of having influence on the treatment they receive is experienced as a way to gain control over their disease management (Quinn et al., 2011). Moreover, our findings revealed that the patients made treatment decisions with the feeling of having no choice. Existing research studies match these findings (Abhyankar et al., 2016; Dellson et al., 2018) by showing the patients' feeling of having no choice was influenced by their perception of receiving treatment as the only real choice because declining treatment meant giving up on life.

Findings from the present study also disclosed that the role of the healthcare professionals affected the decision‐making and that patients had specific expectations regarding the respective roles of the physician and the nurse. Moreover, they experienced the physicians and nurses contributed to separate parts of the conversations. Compared to this, a study by McCullough et al. found that physicians and nurses have distinct but complementary communication roles in the treatment decision‐making process (McCullough et al., 2010). However, in our study the interviewed patients experienced the nurses as being less important than the physicians with regard to treatment decision‐making despite the nurses having been present at all the consultations where the patients were informed about their treatment opportunities. Nevertheless, our findings also showed that patients experienced the nurses as important in relation to talking about existential issues such as managing life with advanced cancer. This might be related to the fact that the nurses were recurring persons in the patients' course of disease, being the ones who provided the continuing treatment and care at the outpatient clinic. Moreover, patients with advanced cancer receive treatment over a long time period and hence, often develop a relationship with the nurses. Existing research literature shows that even though nurses are less noticeable in the process, they have a significant impact on treatment decisions (McCullough et al., 2010). In the mentioned study, nurses described actions with specific patients and a significant role in facilitating patients' decision‐making during care and clinical activities, for example, concerning treatment expectations and reflecting on choices. This indicates that nurses could have an equally important role as physicians in facilitating treatment decision‐making. However, in our study it might have been less noticed because although the patients talked about their experiences with the nurses in general, the interviews tended to focus on the conversation that took place at the consultations when patients were being informed about clinical trial participation.

Regarding the role of the physicians, our findings showed that physicians had a great impact on treatment decisions. This is in line with other research studies describing the substantial role of the physician in treatment decision‐making, which revealed that physicians make preformed decisions without the patients (Ofstad et al., 2014; Salloch et al., 2014) and that patients make decisions based on trust in the physician (Dellson et al., 2018) when physicians make recommendations for treatment (Eggly et al., 2008). Moreover, McCullough et al. (McCullough et al., 2010) found that physicians freely described their own role in treatment decision‐making. In our study, some patients experienced that physicians initiated a talk about existential issues and life coming to an end, probably in order to support the patients in deciding which treatment to choose or whether they prefer not having any treatment at all. However, one of the challenges seemed to be that not all patients were prepared to or interested in talking about their own death and the feelings related to this. Our findings substantiated this, showing that some of the patients tried to ask about prognosis and expressed a wish to talk about life expectancy, whereas other patients said that they did not want to talk about their life coming to an end, indicating that their needs and expectations were very diverse. These variations in patients' needs and preferences are one of the main challenges in patient‐centered communication, which requires a responsive role where healthcare professionals show interest and are receptive to and respectful of patients' needs, values, and preferences (Pluut, 2016; Silverman et al., 2013; Street et al., 2009).

Furthermore, in this context of advanced cancer, communication about cure should not be the pivot of the encounter, and patients may benefit from communication that focuses more on healing than on cure (Hutchinson, 2011). Healing has been described as a transition from anxiety and suffering toward a sense of integrity, completeness, and inner peace. This process requires more conscious communication, with a focus on relational and nonverbal communication. These are skills that must be learned individually by encouraging healthcare professionals to focus more on professional development within existential issues by, for example, offering training that includes reflection on the healthcare professionals ‘feelings and reactions so that they can apply them when caring for the patient (Hutchinson, 2011). This requires systematic training in areas such as active listening, self‐reflection, and awareness of their own nonverbal language, etc. (Krasner et al., 2009; McCormack et al., 2017). These are skills that are essential from the very first step in the decision‐making process, which is to address the patients’ emotions.

Our findings showed that patients particularly experienced that the whole situation seemed to be addressing them as a patient and less as a person, which came to light when the patients described how the decision‐making process seemed to focus on treatment – treatment to maintain hope, treatment to cure the cancer, and information about treatment, and in several cases without involving the context of advanced cancer such as addressing the patients' treatment expectations, what hope is, the difficulty of talking about death etc. In our study, we found that these barriers to involve the context of advanced cancer were associated with both the physicians' role and the patients themselves. Concerning this finding, it is a well‐known discussion among healthcare professionals in general how and whether they should talk with the patients about hope, life, and death. However, our findings also document how emotions influence the decision to be made and therefore can be addressed with advantageous results. According to existing research, this might to a higher degree be accommodated by healthcare professionals with adept communication skills, who are capable of engaging with the patients in focused active listening, exploring the patient's emotional response, by for example catching cues and concerns, and are trained to talk with the patients about both the medical issues and their lives with the disease (Hvidt et al., 2018; Pollak, 2019).

### 4.1 Limitations and strengths

In the present study the vast majority of patients interviewed were women, and the results might have been different had there not been this gender imbalance. Variation in time from consultation to interview might also have had an impact on the patients' experiences, although no differences in the results were notified. The study also has several strengths. The participating patients had different cancer diagnoses, ages, and treatment options. Therefore, they contribute with different aspects of patients' experiences according to clinical trial decision‐making. To reduce memory errors, the patients were interviewed within a few weeks after the consultation where they were informed about their treatment options.

### 4.2 Relevance to clinical practice

The findings from our study indicate that information about clinical trials and decisions about experimental treatment could benefit by being a more integrated part of the individual patient's course of disease. Informing patients about the experimental treatment along with both other treatment options and the option of “no treatment” might clarify that it is a decision between valid treatment options. Moreover, the findings reveal the value of conversations about existential issues and may contribute with a more nuanced way of embracing the palliative patient's life and values when decisions about clinical trials are made. In light of this, it might be advantageous if the physicians worked closer together with the nurses in the decision‐making process so that conversations about existential issues and the nurses' knowledge about the patient were a greater part in the decision‐making process.

## 5 CONCLUSIONS

The study shows that patients with advanced cancer made trial decisions based more on emotions than facts. The decisions about receiving experimental treatment were often based on fear, hope for a cure, and the feeling of having no real choice. The role of the physician had a great impact on the decisions because patients entrusted the decisions to the physician, who also influenced the decisions by the way he/she talked with the patients about their course of disease. The role of the nurse was associated with more existential aspects of life. In the context of advanced cancer, where the effect of the treatment is uncertain and not curative, the decision to participate did not represent all the patient's needs. Dialogue on prognosis and thoughts about how to live life when it is coming to an end should be considered as an important aspect of the decision‐making process.

## AUTHOR CONTRIBUTION

TAG has made substantial contributions to conception and design, acquisition of data, analysis and interpretation of data and drafting the manuscript. RB and JA have made substantial contributions to conception and design, analysis and interpretation of data and been involved in drafting the manuscript and critically revising it. MW and KDS have been involved in drafting the manuscript and critically revising it.

## CONFLICT OF INTEREST

None declared.

## Data Availability

Data sharing not available due to informant anonymity.
